# A General Introduction

**Published:** 1919-05-17

**Authors:** 


### THE NEW DERMATOLOGY.

### I. A General Introduction.

In a recent characteristic address Dr. Leonard Williams declared that "present-day dermatology is a revel of irrelevant terminology," and his remarks concerning dermatologists were even less flattering. Much of his criticism will be endorsed not only by those who have made no special study of skin diseases, but by dermatologists themselves. Nevertheless, he and his fellow-critics must remember that dermatological nomenclature is for the most part a heritage of the days when a long descriptive name in Latin or Greek gave satisfaction to its donor, whereas there is a pleasing tendency among modern dermatologists to study their subject anew in the light of recent pathological research.

With medicine, dermatology may be said to have progressed through three stages—that of clinical description, that of morbid anatomy, and that of bacteriology. It is to the old clinicians that we owe most of the complicated terminology, but their descriptions still remain models of clinical observation: to Unna and Darier we are indebted for much of our knowledge of the morbid histology of the skin; and Sabouraud, perhaps, deserves most credit for the researches that have been made on cutaneous bacteriology. No one of these stages can yet be considered as complete; new facts are still being established by means of accurate clinical observation, particularly with regard to the association of disorders of the skin and its appendages with other diseased states; new methods of histological and histo-chemical research will surely reveal to us much that is now obscure; and we are still far from understanding the exact *rôle* played by bacteria even in some of the commoner diseases of the skin.

In this series of articles an attempt will be made to bring forward some suggestive facts, and to discuss certain theories which throw light on the etiology of eruptions, the significance of which is still but little understood.

If we trace the history of the different views that have been put forward from time to time by the numerous schools of dermatology to explain diseases such as eczema, seborrhoea, the so-called seborrhoeic dermatoses, acne, psoriasis, etc., we see that opinion has ever been divided as to the relative importance of "internal" and "external" factors in the causation of these conditions. By some dermatologists disturbances of the normal processes of digestion and metabolism have been considered all-important, by others stress has been chiefly laid on mechanical or chemical irritation and on bacterial infection of the skin itself.

It is now evident that, perhaps in the majority of skin diseases, both internal and external factors play their part. Even in frankly parasitic affections—both animal and vegetable—the internal factor must be considered; for example, ringworm of the scalp is, practically speaking, a disease of childhood which, at any rate in the small-spored variety, disappears spontaneously at puberty. We can only suppose that some change takes place at this time in the tissues of the scalp which is inimical to the fungus, and Macleod has suggested that the secretion of the thymus gland may be necessary for its growth. Again, the secondary results of scabies and of pediculosis corporis differ greatly in different individuals, though they may be living under precisely similar conditions.

And when we come to consider the various bacterial infections of the skin the internal factor is just as evident; granted that a boil is a folliculitis due to the staphylococcus aureus, we do not know what it is that renders one man's follicles a favourable culture medium, while another's are immune. Cultivations from impetiginised eczema yield a profuse growth of a staphylococcus, indistinguishable by known methods from that which is found in boils; but an eczematous person may never develop boils, and a case of furunculosis may never become eczematous, though the two conditions sometimes co-exist. Experiments have shown that if an emulsion of living staphylococci be rubbed into the skin of the forearm an acute folliculitis results, whereas the filtered toxins of a broth culture of the same organism are capable of producing an eczematous eruption. The latter observation at once suggests that, though liberated toxins from actively growing staphylococci may cause an extension of an eczematous process, there must primarily be present some pathological condition of the skin, in order to enable the organisms to take on active growth.

In recent years an appreciation of the phenomenon of anaphylaxis has enabled us to obtain clearer ideas concerning the etiology of certain types of eruption; much experimental work has already been done in this connection, and most suggestive results have been obtained, but the subject is one of great complexity.

It is well known that certain people show an intolerance or hypersensitiveness to various articles of food, so that the taking of them leads to anaphylactic symptoms, one of which is the occurrence of urticarial, erythematous, and sometimes bulbous or purpuric eruptions on their skin. It is usually held that the substances which act as toxins in this way are of an albuminous nature, but it is possible that the reactions sometimes obtained with non-proteid substances (*e.g.*, certain drugs) are essentially similar in kind. This question will be referred to later.

As examples of foods which may prove toxic to certain people may be mentioned eggs, cereals, nuts, cheese, fish, shell-fish, tomatoes, strawberries, bananas, etc. In a person showing intolerance to one or more of these substances his susceptibility may be tested by applying some of the suspected material—for example, egg albumen or egg yolk—to an area of skin which has been gently scarified (*cp.* Von Pirquet's reaction with tuberculin). A positive reaction is shown by the appearance of an urticarial wheal at the site of inoculation; the local

**The New Dermatology—(*continued*).**

reaction is usually rapid and may last for several hours. Needless to say, control inoculations must be made before any conclusions can be drawn from the test. Apart from articles of food, anaphylactic reactions may be obtained from external irritants (*e.g.*, poison plants and pollen), and from bacterial toxins.

Asthma, of which "hay fever" is a variety, is probably an example of anaphylactic reaction which may be produced by various irritants, such as the emanations from cats, dogs, and horses, the pollen of several different species of plants, some foods, bacterial toxins, and in all probability decomposition products absorbed from the digestive tract. The cutaneous reaction has been successfully used in testing the susceptibility of an asthmatic to a given toxin. For example, he may be tested with the hair of the horse, cat, or dog, with different foods, with dried bacterial cultures, and with pollen. In this connection it will be remembered that the association of urticarial eruptions and asthma is a close one; and Sir Andrew Clark considered the latter to be an urticaria of the bronchial mucous membrane.

It may well be questioned whether these toxic reactions exhibited by certain people to various substances of widely different chemical and physical nature can all be considered as being due to anaphylaxis in the strict sense of the term, but from a practical point of view this is not of great importance. What is important to realise is that many skin diseases are probably of the nature of cutaneous reactions to toxins, some of which act locally and others after absorption from the alimentary canal or from some focus elsewhere in the body. But, although it is known that different toxins may give rise to the same type of cutaneous reaction—for example, an eruption of erythema multiforme may result from the injection of a foreign serum, from a drug, or from the toxin of a haemolytic streptococcus—different people react to the same toxin in different ways. Indeed, it is to a certain extent possible to classify persons into groups according to their particular reaction to toxic substances, and one is even able to predict in a given person in what way he will react to such substances; thus, one who is subject to urticaria will usually react in this way to a variety of irritants—both external and internal—whereas exposure to these will in another lead to an outbreak of eczema.

In the light of these considerations we may now consider in detail the etiology and rational treatment of some of the more familiar diseases of the skin.

(*To be continued.*)

